# The 3.3 Å structure of a plant geminivirus using cryo-EM

**DOI:** 10.1038/s41467-018-04793-6

**Published:** 2018-06-18

**Authors:** Emma L. Hesketh, Keith Saunders, Chloe Fisher, Joran Potze, John Stanley, George P. Lomonossoff, Neil A. Ranson

**Affiliations:** 1 Astbury Centre for Structural Molecular Biology, School of Molecular & Cellular Biology, Faculty of Biological Sciences, University of Leeds, Leeds, LS2 9JT UK; 2 Department of Biological Chemistry, John Innes Centre, Norwich Research Park, Colney, Norwich NR4 7UH UK

## Abstract

Geminiviruses are major plant pathogens that threaten food security globally. They have a unique architecture built from two incomplete icosahedral particles, fused to form a geminate capsid. However, despite their importance to agricultural economies and fundamental biological interest, the details of how this is realized in 3D remain unknown. Here we report the structure of *Ageratum* yellow vein virus at 3.3 Å resolution, using single-particle cryo-electron microscopy, together with an atomic model that shows that the N-terminus of the single capsid protein (CP) adopts three different conformations essential for building the interface between geminate halves. Our map also contains density for ~7 bases of single-stranded DNA bound to each CP, and we show that the interactions between the genome and CPs are different at the interface than in the rest of the capsid. With additional mutagenesis data, this suggests a central role for DNA binding-induced conformational change in directing the assembly of geminate capsids.

## Abstract (web summary)

Geminiviruses are an important plant pathogen that causes large food crop losses globally. Here the authors describe a high resolution cryo-EM structure of the *Ageratum* yellow vein virus and reveal the molecular details of how a single capsid protein sequence can adopt the different conformations needed to build that geminate capsid.

## Introduction

Geminiviruses are thought to have been responsible for an unseasonal change in the appearance of the summer foliage in plants that was described in the poetry of the Empress Kōken in Japan in 752 AD^1^. Geminiviruses are thus probably the cause of the first available record of a viral disease in plants. In modern times, diseases associated with geminiviruses include maize streak disease and cassava mosaic disease in Africa; golden mosaic disease of beans in the Americas; tomato yellow leaf curl disease across much of the globe; and cotton leaf curl disease across India and Pakistan. The causative agents of many of these diseases are *Begomoviruses*, a genus in the *Geminiviridae*, which have circular, single-stranded^2^ DNA genomes of total size ~2.7 to ~5.4 kb. The genetics of *Begomoviruses* are complex. Many have bipartite genomes, consisting of two separately encapsidated DNAs (DNA-A and B, each of ~2.7 kb). Other *Begomoviruses*, such as *Ageratum* yellow vein virus (AYVV) studied here, have a single genomic DNA (DNA-A, ~2.7 kb), along with an array of satellite DNAs (α & β, each of ~1.3–1.4 kb). β satellite (DNA-β), whose replication is totally dependent upon gene functions encoded within DNA-A, encodes a gene which is essential for AYVV infectivity in its natural host plant^3^. In contrast, α satellite DNA (DNA-α) encodes a *Rep* gene responsible for its own replication^4^. The basic requirement for a functional geminivirus capsid is thus that it must be able to encapsidate a single, 2.7 kb circular, single-stranded DNA molecule. The closest plant virus relatives of the geminiviruses are the nanoviruses, which have multipartite, single-stranded DNA genomes that are separately encapsidated and are ~1 kb in size^5,6^. Each such DNA molecule typically encodes a single gene product. Geminiviruses therefore appear to be able to package much larger amounts of DNA than nanoviruses, and to achieve this, they have evolved a unique capsid structure. Whereas nanoviruses have *T* = 1 icosahedral capsids of ~18 nm in diameter (although no detailed structures are available), geminiviruses are formed from two such isometric particles, or ‘hemicapsids’, fused to form a geminate particle (18 × 30 nm) which gives the virus family its name^2,6^. Until recently, no high-resolution structure for a geminivirus was available to describe how this architecture was realized. However, low-resolution cryo-EM studies on maize streak virus (MSV)^7^ and African cassava mosaic virus (ACMV)^8^, combined with homology modeling, suggested that the geminivirus coat protein (CP) adopts a structure similar to that of satellite tobacco necrosis virus (STNV), a single-stranded RNA virus^7^. Indeed, based on an ~25 Å resolution cryo-EM structure of MSV, a model for the geminate interface was proposed with an α-helix from each subunit (found in the STNV CP) extending across the geminate interface^7^. STNV also has a much smaller genome than a geminivirus (~1250 vs ~2700 nucleotides), so geminate capsids again can package more genetic material than a simple *T* = 1 particle, despite encoding a single CP sequence. This is a similar leap in packaging capacity to that made by other viruses in the transition from *T* = 1 to *T* = 3 capsids described by Caspar & Klug in classical quasi-equivalence theory^9^. However, it seems unlikely that this arose from a simple expansion of the DNA genome beyond that which can be encapsidated within an isometric particle, as in such cases, aberrant structures with multiple, concentric CP shells have been observed^10,11^. A very recent structure of ACMV at 4.2 Å resolution^12^ has begun to reveal details of how the geminate structure is built, proposing that alternating conformations of an N-terminal segment could be a major determinant of the geminate interface. However, the resolution of this structure was insufficient to describe the interface between the two ‘hemicapsids’ in atomic detail, and it contains no interpretable density for the genomic ssDNA or the interactions it makes with the capsid.

Our current knowledge of geminivirus structure thus leaves two questions of fundamental biological interest unanswered. Firstly, how does a single CP accommodate the different conformations required to build a geminate particle? Secondly, no empty geminivirus particles have ever been reported, implying that DNA binding is intimately linked to capsid assembly; but how is DNA recognized and packaged? Here we present the cryo-EM structure of AYVV at 3.3 Å resolution. The structure shows that the capsid is built from three distinct conformations of a single CP, and that these conformational differences facilitate the formation of the interface between hemicapsids. We also show details of single-stranded DNA binding and propose a model for geminivirus capsid assembly.

## Results

### Structure of AYVV

**Fig. 1:**
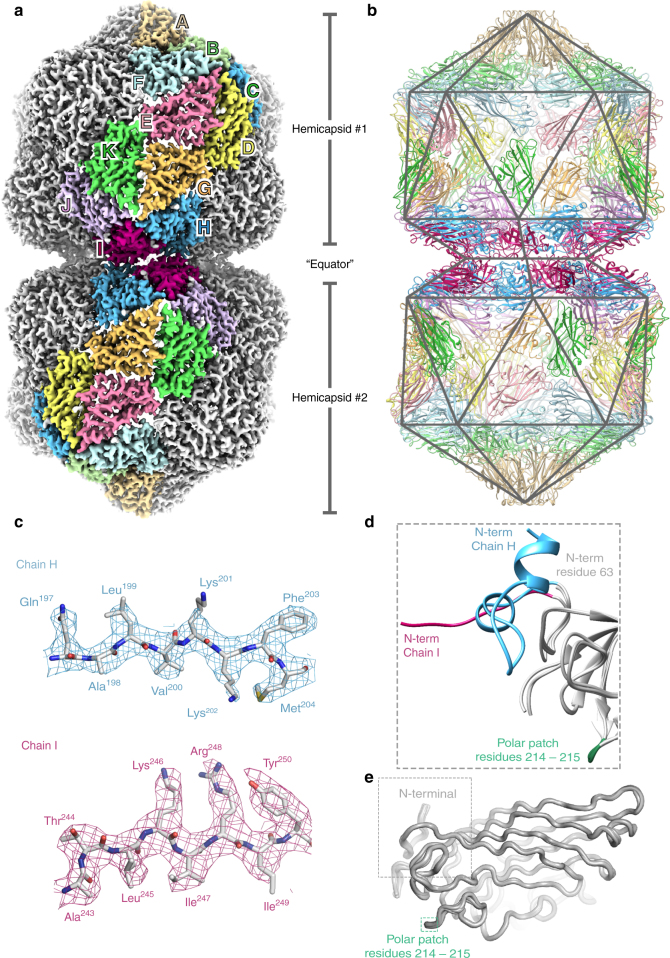
The structure of *Ageratum* yellow vein virus. **a** EM density for AYVV at 3.3 Å resolution. The density is segmented and colored to highlight the 11 unique copies of the AYVV CP in the D5 asymmetric unit (labeled A-K). **b** Complete atomic model for all 110 subunits in the capsid, with a polyhedral cage showing the symmetry of the particle. The fivefold symmetry axis is vertical and through the center of the particle in this view. **c** Representative regions of EM density with the corresponding section of atomic model. **d** A “zoomed in” view of the N-terminal segment of all 11 unique CPs aligned. The N-terminal of chain I, chain H and the remaining 9 CPs is highlighted. The position of a polar patch (residues 214–215) is also shown. **e** Alignment of residues 64–256 from all 11 unique copies of the CP. The RMSD between structures is ~0.2–0.3 Å. The position of the N terminal and the polar patch of residues is indicated

To address these questions, we determined the structure of AYVV using single particle cryo-EM (Fig. 1). AYVV (and, indeed, the majority of the *Begomoviruses*) are phloem-limited and consequently are not mechanically transmissible, so we used *Agrobacterium* transformed with a plasmid containing a partial tandem copy of AYVV DNA-A^13^ (see Methods), to agroinfect^14^ *N. benthamiana* plants (Supplementary Fig. 1). Geminate particles were purified from symptomatic leaves (~2 mg/Kg of fresh leaf tissue) and a cryo-EM dataset collected (see Methods). Structure refinement with D5 symmetry, yielded a density map at 3.3 Å resolution (Fig. 1a and Supplementary Fig. 2), and an atomic model for the CP layer was built and refined (see Supplementary Table 1), starting from a homology model of the STNV CP (see methods). The capsid is composed of 110 copies of the CP, and we built and refined a complete D5 asymmetric unit of 11 unique chains (chains A-K) (Fig. 1b–e). As described previously^7,8,12^, each hemicapsid is formed from a *T* = 1 particle from which a penton of CP subunits is missing. The resulting facets on each hemicapsid associate to form the geminate particle (Fig. 1a). The body of the CP is similar to that of STNV and many other viruses, consisting of a jelly-roll fold with two twisted, 4-stranded β-sheets. This core domain structure is identical in all 11 chains in the asymmetric unit (Fig. 1e).

### The structure of the CP N-terminal conformations

**Fig. 2:**
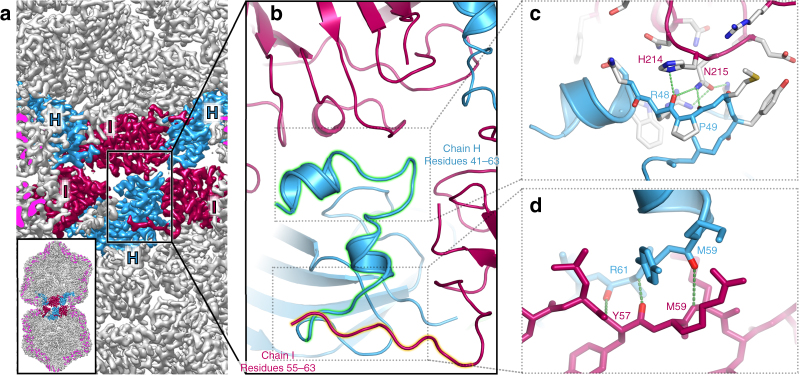
Building a geminate capsid. **a** Rear half of the EM density for AYVV at 3.3 Å resolution. The cut surface of the EM density is colored bright magenta for clarity. Three CP subunits from each hemisphere are colored as in Fig. 1. In the top hemisphere, these are H-I-H (blue-maroon-blue) and in the bottom hemisphere these are I-H-I (colored maroon-blue-maroon). **b** Zoomed-in view of the interactions that form the interface. A helix-loop-helix motif comprising residues 40–63, becomes ordered only in subunit H (green halo), whilst an extra segment (residues 55–63) in subunit I also becomes ordered (orange halo) but in a different conformation to that seen by the same residues in chain H. **c** Details of the interactions across the equatorial interface. The loop in the helix-loop-helix motif makes H-bonding and van der Waals interactions with a patch of polar residues (214–215) on the opposing subunit. This patch is normally exposed on the surface of the subunits that form the isometric parts of the capsid (see Supplementary Fig. 4). **d** Details of the interaction between the two different N-terminal conformations. Effectively a beta-strand interaction is formed from backbone H-bonding of residues 59–61 in H (blue) and residues 56–59 in I, which helps to stabilize the ring of H & I subunits within a hemisphere

The different conformations needed to build a geminivirus arise through differences in the N-terminal domain of the CP, rather than in its core fold. Many ssRNA plant viruses, including STNV, have positively-charged, N-terminal domains that play roles in binding RNA (e.g. refs. ^15,16^), and which are disordered in all structures (both X-ray and EM) that have been determined so far. A similar phenomenon is observed here for this ssDNA virus. The N-termini of the CP of AYVV are considerably longer than those seen in STNV, presumably at least in part because the first ~20–22 residues constitute the nuclear localization signal which is required for a DNA virus. However, they share the common theme seen in all N- and C-terminal extensions to viral CPs that project into the encapsidated space, in that they are strongly positively charged (Supplementary Fig. 3). In the subunits that comprise the majority of the hemicapsids, all subunits have a common first resolved residue (residue 63). However, additional amino acids are resolved in the subunits that make the equatorial interface (chains H (blue) and I (maroon)) (Fig. 2a). The biggest change is in subunit H, where an extra 23 residues are resolved as a helix-loop-helix motif (first ordered residue: 40) (Fig. 2b). By contrast in chain I, an extra 8 residues (first ordered residue: 55) become ordered, but in an extended conformation that is different to that seen for the same residues in chain H (Fig. 2b). The N-terminal extensions in subunits H and I are positively charged (Supplementary Fig. 3). These two new segments play very different roles in stabilizing the capsid. The helix-loop-helix motif from chain H is a major component of the equatorial interface (Fig. 2c), making a series of H-bonding interactions to a patch of predominantly polar residues in the body of the CP subunit across the interface (residues 214 & 215 in chain I; see Figs. 1d, e and 2c, and Supplementary Fig. 4), which is surrounded by van der Waals interactions. This polar patch is normally solvent-exposed on the surface of the virus in the subunits that form the hemicapsid (see Supplementary Fig. 4). The ordered segment from chain I extends across to stabilize the base of the helix-loop-helix motif in an adjacent H subunit, presumably stabilizing the ring of equatorial subunits (Fig. 2d). This interaction is mediated by backbone hydrogen bonding; essentially, a short (~3 residue) domain-swapped, antiparallel β-strand interaction is formed. The geminate particle therefore critically relies on a previously disordered segment of the N-terminus of the CP acquiring two different conformations to build the equatorial interface, which local resolution analysis suggests is marginally the most ordered part of the structure (Supplementary Fig. 5).

### The role of the N-terminal extension in chains H and I

Our structure makes a simple functional prediction: that interactions between chain H, and chain I are critical to particle assembly, and that disrupting these interactions might favor single rather than geminate particle formation. However, the geminate capsid is required to encapsidate AYVV’s genomic DNA, the 2.7 kb DNA-A; destabilization of the geminate interface could therefore lead to degradation of the genomic DNA in vivo and abolish particle formation of all types.

**Fig. 3:**
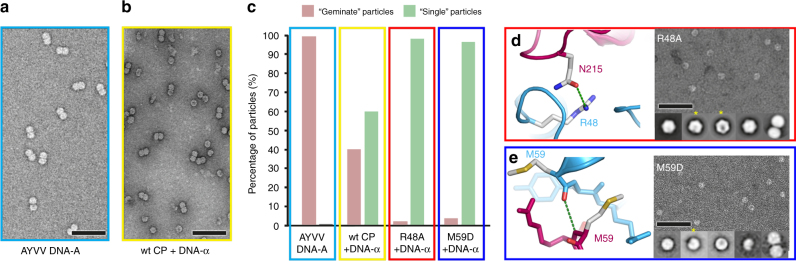
In vivo assembly assay for genome encapsidation. Representative negative stain micrographs of particles produced by infiltration with AYVV DNA-A (**a**) or co-infiltration with AYVV CP and DNA−α (**b**). Relative composition of “geminate” particles (pink in bar chart) compared with “single” particles (green in bar chart) from AYVV (**c**), and co-infiltrations of AYVV CP, R48A, and M59D, each with DNA−α (**c**). Representative negative stain micrographs and 2D class averages are shown for R48A variant (**d**) and M59D variant (**e**). The yellow asterisk highlights 2D class averages which appear to be “single” VLPs with a missing pentameric capsomer. Error bar is 100 nm

We therefore exploited our knowledge of geminivirus genetics to establish an in vivo assay for encapsidation and assembly. AYVV infection of *Ageratum conyzoides* requires its 2.7 kb genomic DNA-A, together with its 1.3 kb β-satellite DNA, to give the classic yellow vein symptoms on this host^3^. However, in experimental hosts such as *N. benthamiana*, leaf curl symptoms are evident with inoculations of DNA-A alone. In the natural host, this “A + β” infection complex is always associated with DNA**-**α (again, ~1.3 kb in length). DNA**-**α encodes a *Rep* gene that facilitates its own replication, but is encapsidated by the CP encoded by DNA-A, and it is this encapsidation that is essential for vector transmission and ultimately for maintenance of the DNA**-**α itself. We therefore took advantage of this by over expressing geminiviral CP, in the absence of a geminivirus infection, and at the same time introduced the self-replicating DNA**-**α into the infiltration system. The result was the formation of a mixture of “geminate” and “single” virus-like particles (VLPs). Importantly no VLPs are generated by the expression of geminivirus CP alone, strongly indicating that the provision of circular ssDNA is essential for capsid formation irrespective of whether “geminate” or “single” VLPs are generated. In the presence of wildtype CP and DNA**-**α, the ratio of geminate to single VLPs is ~40:60, compared to essentially 100% geminate particles for the wildtype virus (Fig. 3a–c). We then made two mutations within the CP to probe the effect of interface destabilization. Firstly, we made R48A, which we predicted would disrupt the H-bonding between to the ‘top’ of the helix-loop-helix motif and chain I in the opposite hemicapsid (shown in Fig. 2c). We also made M59D, which we predicted would disrupt the main chain H-bonding interaction between chain I and the base of the helix-loop-helix motif in chain H (shown in Fig. 2d). This region is stabilized by backbone hydrogen bonds between residue M59 in the two chains, as part of the short β-strand interaction described above, and we rationalized that the introduction of a negative charge in this region would disrupt this specific interaction. For both mutations, an almost complete switch from geminate to single particles was observed with DNA-α (Fig. 3c–e), confirming the importance of these regions for geminate capsid formation. Strikingly for both the R48A and M59D variants, some of the resulting ‘single’ VLPs appear to have a missing pentameric capsomer in negative stain EM image averages (Fig. 3d, e).

### Interactions between the genomic DNA and CP subunits

**Fig. 4:**
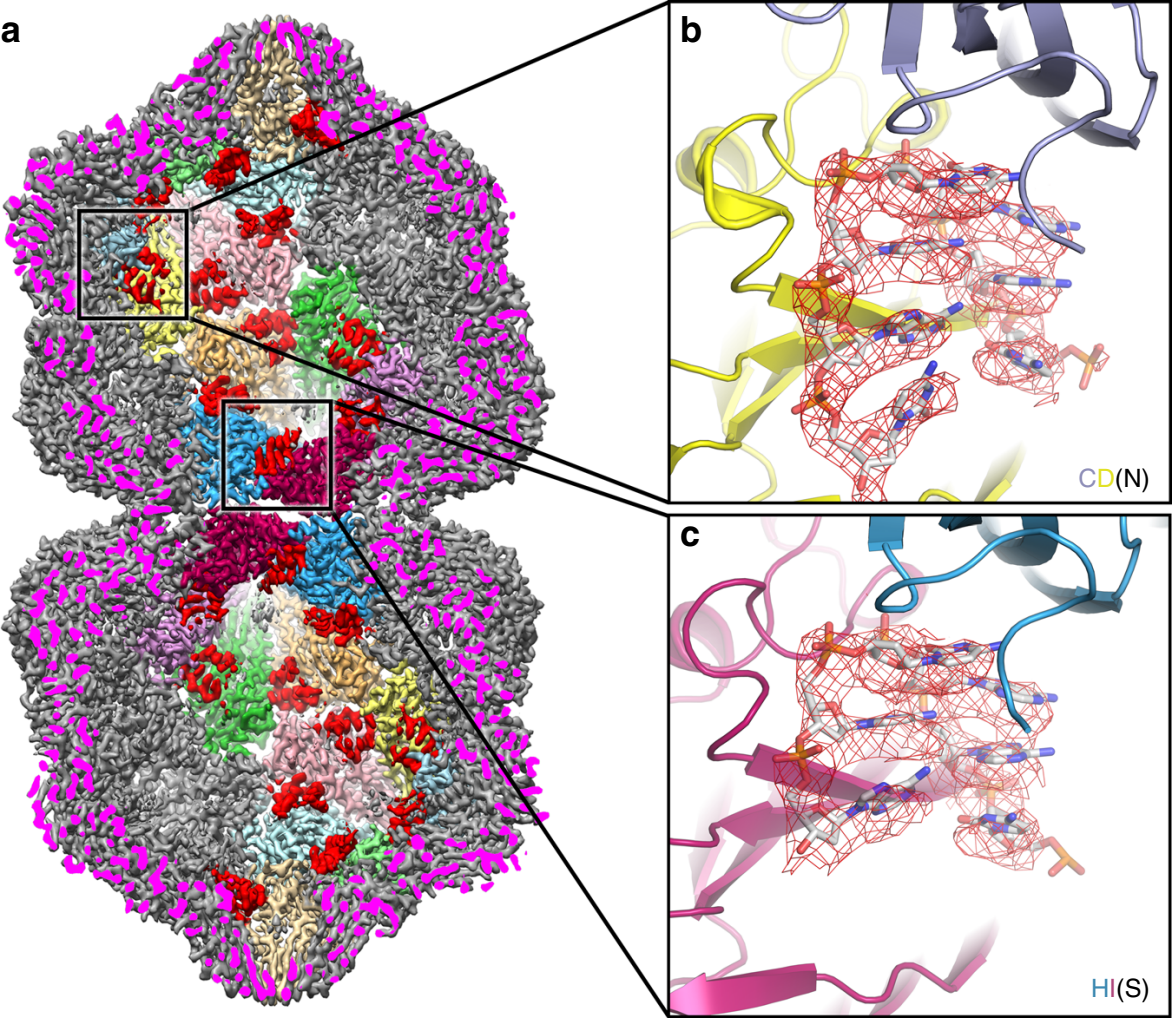
Single-stranded DNA binding. **a** Rear half of the EM density for AYVV at 3.3 Å resolution. The cut surface of the EM density is colored bright magenta for clarity. Two D5 asymmetric units are shown at the back of the capsid (colored as in Fig. 1a). High-resolution density corresponding to ssDNA is in red. **b** The EM density for 7 nucleotides of DNA is shown as a red mesh and the modeled atomic coordinates for CPs C and D and DNA stem loop N (CD-type DNA) is shown. These 7 nucleotides and the mode of binding to the neighboring subunits is identical for the majority of the CPs (i.e. A-G & I-K). At the interface (i.e. CPs H and I) these interactions are different, **c** shows 6 nucleotides, stem loop S, and the neighboring CPs H and I (HI-type DNA), depicted as described in **b**

**Fig. 5:**
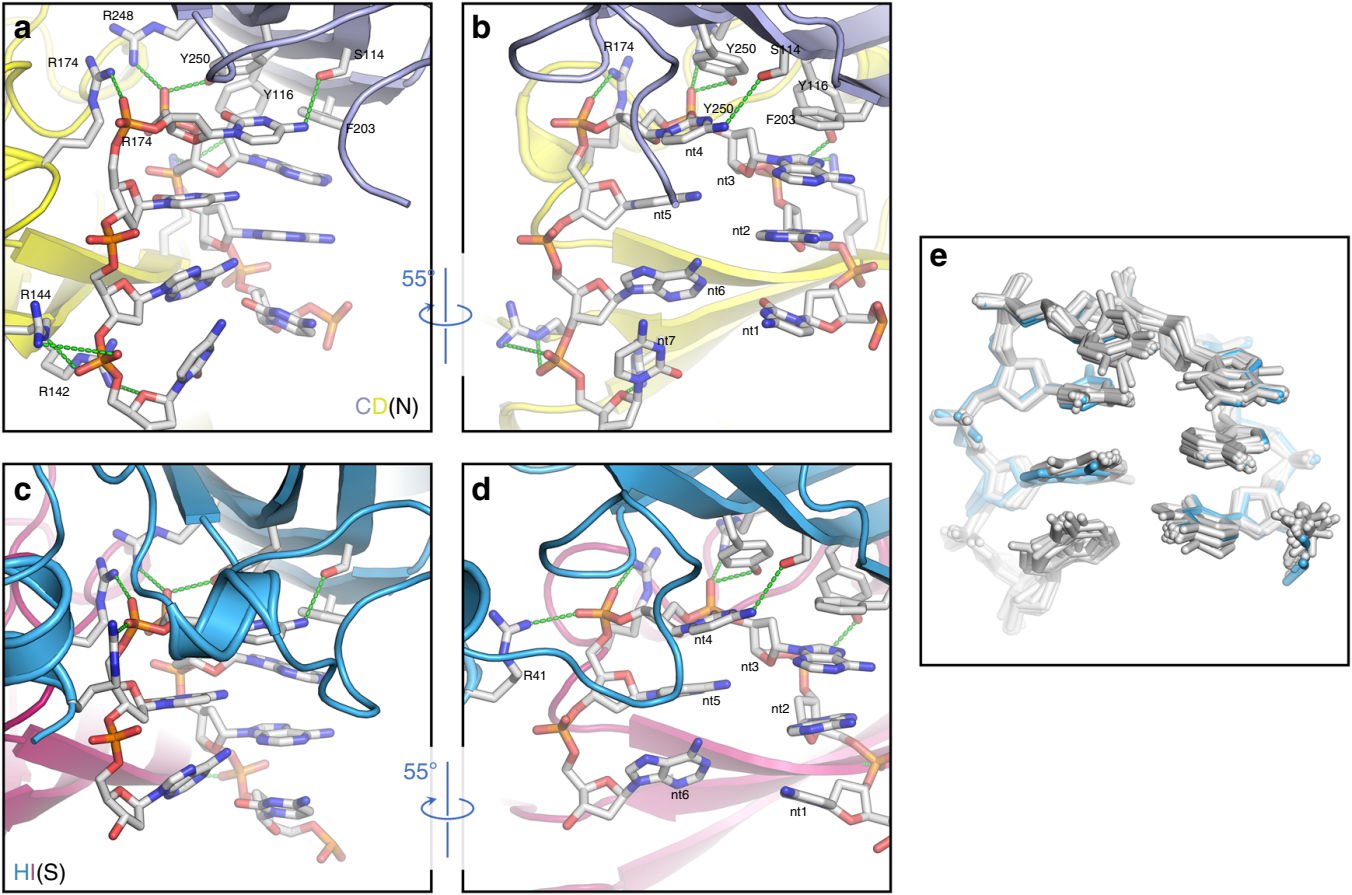
Details of interactions between DNA and CP. The details of the interactions between CP subunits and bound DNA. **a** The atomic model of “CD-type” DNA (i.e. DNA:CP interactions in the majority of the capsid (CPs A-G & I-K)) and the neighboring CPs (C and D). **b** as in **a** rotated 55 degrees as indicated. **a**, **b** show the interactions between polar sidechains and both the DNA bases and backbone at positions 2, 3, and 4, along with a base stacking interaction between F203 and the base of nucleotide 4. **c**, **d** The atomic model of “HI-type” DNA (i.e. DNA:CP interactions at the interface) and the neighboring CPs (H and I). At the interface, we see an extra interaction formed from the extreme N-terminus of the ordered density (residue 41) and the fifth nucleotide in the stem loop, which appears to stabilize the ordered N-terminal helix-loop-helix domain (**c**, **d**). **e** The overall conformation of the DNA stem-loop in each position is essentially identical. In **e** all chains are colored gray, except for H (blue)

A geminate capsid thus requires three distinct conformations, which although not classically ‘quasi-equivalent’ as they do not map to positions within an icosahedral lattice, fulfill a similar role in allowing a bigger capsid to be built. However, a fundamental question remains: how does a CP subunit ‘know’ what position it occupies within the overall architecture of the capsid? One solution would be for the conformation to be specified through interaction with the genome, as has been shown for single-stranded RNA viruses^17,18,19^. We therefore looked at the extensive density in our EM map that was unoccupied by the atomic model for the CP (Fig. 4; Supplementary Movies 1 and 2). Density is present beneath every CP in the capsid, consistent with an interaction with a ssDNA stem-loop. The map was of sufficient quality to build 7 nucleotides into the density beneath each subunit, except for subunit H at the interface where there are 6. This density is as strong as that for the CP, suggesting that occupancy is high, but owing to the D5 symmetry averaging applied, we cannot definitively say that all sites in the capsid are occupied. However, assuming that they are, our structure resolves ~28% of the genomic DNA molecule at high resolution (Figs. 4 and 5). There is some suggestion from the density, which is slightly different for each of the 11 DNA chains in the asymmetric unit, as to whether each position is a purine or pyrimidine, and thus we have built a very tentative consensus sequence of YRRYYRY into the density (where Y represents a generic pyrimidine, and R a generic purine; using adenine for R, and cytosine for Y).

For each DNA chain in the body of the capsid (i.e. chains A-G and J-K), and for chain I, interactions are made between one CP by four residues (S114, Y116, R248, and Y250) to the top of the bound stem loop (nucleotides 3 and 4) (between the DNA and the blue subunit in Fig. 5a, b; Supplementary Movie 3). Additional contacts are made between arginine residues in a second CP subunit (R142, R144 & R174) (the yellow in Fig. 5a, b; Supplementary Movie 3) and the DNA backbone at the 3′ end of the bound stem loop (nucleotides 5 & 7). In chain H, we again see a difference compared to the other chains. At the top of the stem loop, the interactions remain unchanged (between the DNA and the blue subunit in Fig. 5c, d; Supplementary Movie 4). More significantly, a new interaction occurs between the backbone of nucleotide 5 and R41, at the extreme N-terminus of the helix-loop-helix motif in chain H. This motif forms the equatorial interface, and presumably the interaction with DNA stabilizes this structural element. Despite this, overall the conformation of the bound DNA is very similar at each position, including at the equatorial interface (Fig. 5e).

To test the importance of DNA binding, we also made mutant R41A, to disrupt the interaction between R41 and the DNA backbone. When this was expressed alongside DNA-α, no particles were recovered from leaves, suggesting that DNA binding and/or assembly is severely compromised, as expected from our predicted role for R41.

## Discussion

The structure of AYVV presented here helps to answer several long-standing questions in geminivirus biology. Early cryo-EM structures at low resolution (25–30 Å)^7,8^, together with the pioneering work by Liljas and colleagues to determine the crystal structure of STNV^20^ established a basic architecture for a geminate capsid, and suggested that it would be built from viral CPs with an STNV-like, jelly-roll fold. McKenna and colleagues^7^ suggested an initial model, where the geminate interface was formed by conformational switching of the CPs at that interface, allowing the exchange of helices, tilted relative to the plane of the interface, between CPs. The STNV structure upon which this model was based does indeed have an N-terminal α-helix of varying length^15,20^. Recent work on ACMV^12^ at much higher resolution (~4.2 Å) moved this tentative model forward, by suggesting that alternate conformations of subunits occurred at the geminate interface. The structure at 3.3 Å resolution presented here confirms this, and now provides molecular details of this conformational switching, the conformations adopted, and the interactions made. Two new conformations of portions of the N-terminus are observed in chains H and I at the geminate interface – one (chain H) in a helix-loop-helix motif, that forms an integral part of the geminate interface and the other (chain I) in an extended conformation that stabilizes chain H, via a short, domain-swapped β-strand interaction. These conformations are not compatible with the previous ‘helix-swapping’ model proposed for MSV; no discrete bridges of density are observed. Rather, the helix-loop-helix motif in our structure forms a relatively flat surface and thus a more intimate interface, with the centers of mass of the two hemicapsids moved closer together than has been proposed for MSV.

The structure presented here also demonstrates a role for DNA binding in specifying protein conformation. The recent 4.2 Å structure of ACMV resolves no ordered DNA^12^, but tentatively assigns amorphous density beneath the fivefold axis of each capsomer to DNA. We see very similar features in our map; however, this density disappears during map sharpening, which removes low resolution information from the reconstruction. This strongly suggests that, irrespective of whether this density corresponds to the N-terminal domains of the CP, genomic DNA, or a mixture of the two, no ordered structure exists at these positions in a geminivirus capsid. The structure presented here does, however, resolve large amounts of the genomic single-stranded DNA at high resolution, at a position beneath each CP subunit. This is not unprecedented for single-stranded DNA viruses, as structures of parvoviruses resolve longer segments (up to 11 bases of ssDNA in canine parvovirus)^21^. However, given that the parvovirus genome is rather larger (~5 kb), this represents only ~13% of the genomic DNA, compared to ~28% in this 3.3 Å structure of AYVV. An examination of the unsharpened map, suggests that even a 2.7 kb genomic DNA would fill the entire available space within a particle, suggesting different genomic components (~2.7 kb) and satellite geminivirus DNAs (~1.4 kb) are likely to be encapsidated separately.

The DNA loops resolved in the AYVV structure sit beneath each CP, but make interactions with a second, neighboring CP, immediately suggesting a role for the DNA in directing the assembly of the particle. This suggestion is then bolstered by the observations of DNA binding at the geminate interface, with a specific interaction between R41, at the extreme N-terminal end of the helix-turn-helix motif, and the DNA backbone of nucleotide 5. We therefore see a specific interaction, between a uniquely ordered protein segment at the interface and the DNA, which when disrupted by mutagenesis abolished capsid formation, suggesting a role for DNA in directing the assembly of the particle. Despite this, overall the conformation of the 6 nucleotides found at every position in the bound DNA are strikingly similar, including at the equatorial interface (Fig. 5e). This in turn suggest that perhaps the spatial arrangement of loops rather than a fundamentally different mode of binding may specify the geminate capsid. However, our current data do not allow us to propose a consensus DNA sequence for binding with sufficient confidence to determine the number and/or position of binding sites within the genome.

**Fig. 6:**
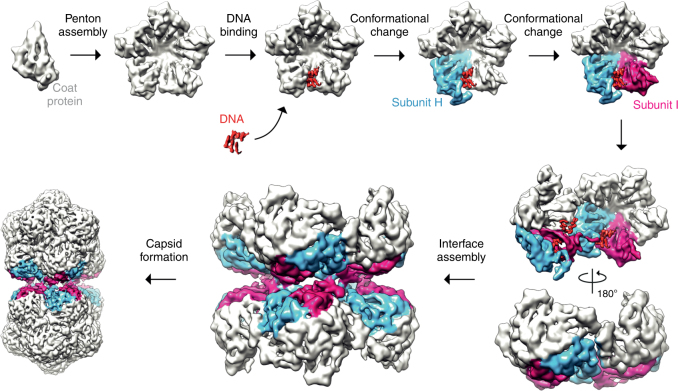
A model of geminate particle assembly. Five CP subunits assemble into a penton, the building block of the geminate capsid. A DNA stem loop specifically binds to the N terminus of a CP subunit triggering 23 amino acids in the N terminus to become ordered and adopt the chain H conformation (blue). The neighboring subunit also undergoes a conformational change where eight amino acids in the N-terminal become ordered and adopt chain I conformation (maroon). The combination of these conformational changes allows the interface of a geminate capsid to assemble at the equator. Finally, the hemicapsids are assembled by addition of CP pentons

These data together with previous observations in other viral systems suggest a simple model for geminate particle formation (Fig. 6). Given that each hemicapsid is missing a single penton, and that the interface between hemicapsids is pentameric, it seems plausible that the fundamental building block or ‘capsomere’ of the geminate capsid is a penton of CP subunits. The penton as capsomere has been suggested or shown for a broad range of viruses with different genome types, including animal- and plant-infecting viruses of the order picornavirales^22–25^ with ssRNA genomes, and indeed geminiviruses, where negative stain EM has shown that disassembly of ACMV occurs to isolated  pentons^26^. Assembly of these ‘capsomeres’ appears to then be driven by DNA binding, explaining both the observation that no empty capsids have ever been reported for any geminivirus, and that specific disruption of an interaction between DNA and CP (via R41A) abolishes capsid formation. Again, this fits well with observations from other systems. For example, a role for the ssDNA genome in canine parvovirus assembly has previously been postulated^27^, and more recently the assembly of beak and feather disease CP into higher order assemblies has been convincingly demonstrated to be stimulated by ssDNA binding^28^. The role of ssRNA is more solidly characterized, with structures of ribonucleoprotein complexes for viruses such as Pariacoto virus^29^ and STNV^15^ that suggest a role for nucleic acid in templating assembly. We speculate that a stem-loop that is different in either sequence or structure, promotes the conversion of a CP into the chain H conformation. The unique stem-loop structure found within the origin of replication for all known geminiviruses^13,30^ is a plausible candidate for this role, but we have no experimental evidence to support this idea at present. Concurrently, either protein-protein and/or protein-DNA interactions, promotes a neighboring subunit to adopt the chain I conformation. It is important to note, however, that this order is speculative. At this point however, the fundamental building blocks for a geminate capsid would be present, and an appropriate spatial distribution of stem loops would promote the assembly of these building blocks to form the capsid, with other nascent stem loops binding to non-equatorial positions in the high local concentration caused by encapsidation. Whilst speculative, this model provides a simple explanation to a long-standing puzzle as to how geminate particles are formed that is consistent with the high resolution structural observations of protein-protein and protein-DNA interactions described herein. A key goal of our future studies will be to validate this model by determining the sequence of the DNA bound to the AYVV CP, and thereby the number or position of these loops within the genomic DNA.

## Methods

### Infection of plants with AYVV DNA-A

The construction of pHNBin419 containing a partial tandem copy of AYVV DNA-A has been described^13^. Cultures of transformed *Agrobacterium tumefaciens* strain GV 3850 harboring this plasmid were used to mechanically inoculate the stems of two-week-old *Nicotiana benthamiana* using the method previously described^31^. Inoculated plants were subsequently allowed to grow at 25 °C in an insect-proof glasshouse for 20–25 days with 16 hours supplementary lighting.

### Expression of wild-type and specific mutants of AYVV CP

Oligonucleotides KS37 and KS38 (Supplementary Table 2) were used with pHNBin419 DNA as template and Phusion polymerase (New England Biolabs) for PCR to obtain DNA encoding the AYVV CP that was subsequently cloned via the BP reaction into pDONR207 (Invitrogen). A sequenced-verified clone was transferred via the Gateway LR reaction into pEAQ-*HT*-DEST1^31^ to yield pEAQ-*HT*-D1-AYVVCP. The Agilent “QuikChange Primer Design” website, http://www.genomics.agilent.com/primerDesignProgram.jsp was used to design primer pairs to introduce mutations into the AYVV CP in pEAQ-*HT*-D1-AYVVCP at residues 41 (R to A: KS125P and KS126P), at residue 48, (R to A: KS127P and KS128P), and at residue 59 (M to D: KS129P and KS130P); Supplementary Table 2. pEAQ-*HT*-D1-AYVVCP was used as the DNA template for each reaction as described in the GeneArt site-directed mutagenesis system (Invitrogen). The sequences of the wild-type and mutated clones were verified prior to transforming *Agrobacterium tumefaciens* strain LBA 4404 for transient expression in plants. The AYVV satellite DNA**-**α partial repeat clone, pBin AYVV 1/7, was pressure co-infiltrated with pEAQ-*HT*-D1-AYVVCP or its mutants as described previously^32^.

### Isolation of virus and virus-like particles

Symptom-displaying leaves were harvested and ground at 4 °C in 3 volumes (1 g/3 mL) of extraction buffer (100 mM sodium phosphate buffer pH 6.0, 10 mM Na_2_SO_3_, 0.1% (v/v) 2-mercaptoethanol, 0.5% (v/v) Triton X-100, 0.1% (w/v) Driselase (Sigma-Aldrich)), supplemented with EDTA-free protease inhibitor tablets (Roche), 1 tablet/100 mL. After overnight incubation at 4 °C with constant stirring, the mixture was squeezed through Miracloth (Merck Chemicals) and clarified by centrifugation at 11,500×*g* for 20 min. To the resulting supernatant, one quarter volume of chloroform was added and mixed for 10 min and the phases separated by centrifugation at 12,000×*g* for 20 min. One quarter volume of a 1 M NaCl, 20% (w/v) PEG solution was added to the recovered aqueous phase and the resulting mixture was stirred overnight at 4 °C. Precipitated virus particles were collected by centrifugation at 16,500×*g* for 20 min. The pellet was resuspended in 100 mM sodium phosphate buffer, pH 7.0 and the solution clarified by centrifugation at 27,000×*g* for 20 min. To the supernatant, Cs_2_SO_4_ was added to a final concentration of 36% (w/v) and the solution centrifuged at 247,103×*g* for 24 hours at 5 °C. Gradients were fractionated into 0.5 ml fractions and the fractions containing viral CP were identified by electrophoresis in 12% (w/v) NuPAGE MOPS-buffered gels (Life Technologies) followed by staining with Instant Blue dye (Expedeon Ltd). Viral CP identity was confirmed by subsequent MALDI-TOF analysis of the gel band containing the putative CP (John Innes Centre, platform facility). Selected fractions were dialyzed overnight at 4 °C against 100 mm sodium phosphate buffer pH7.0 and subsequently centrifuged at 210,000×*g* through a 20–60% (w/v) step sucrose gradient for 3 hours at 5 °C. Gradients were again fractionated, and the virus containing fractions identified by gel electrophoresis. After overnight dialysis against 100 mM sodium phosphate buffer, pH7.0, the samples were concentrated using Amicon Ultra-15 100,000 MWCO spin concentrators as described by the manufacturer.

### Negative stain electron microscopy

Negative stain EM grids were produced by applying 3 μl of particle preparations on to carbon- coated copper grids. Excess liquid was blotted away, the grids were washed in water twice and once in 2% (w/v) uranyl acetate, excess liquid was removed and grids were air-dried. The grids were imaged using either an FEI Tecnai F20 EM fitted with an FEI CMOS camera or FEI Tecnai 12 EM fitted with a Gatan US4000 (Astbury Biostructure Laboratory, University of Leeds). The number of particles in each analysis were as follows: WT virus 1,489 particles, WT CP and DNA-α 4,164 particles, R48A mutant 16,015 particles, M59D mutant 12,639 particles.

### Cryo-EM

Cryo-EM grids were prepared by applying AYVV to 400 mesh grids with a supporting carbon lacey film. The lacey carbon was coated with an ultra-thin carbon support film < 3 nm thick (Agar Scientific, UK). Grids were glow-discharged for 30 seconds prior to applying the sample (easiGlow, Ted Pella). To increase the number of particles that adhere to the carbon, 3 μl of the sample was incubated on the grid for 5 min. The majority of the liquid evaporated during the incubation, but at no point was the grid allowed to dry out; this was repeated three times. The final 3 μl was blotted immediately using a Leica EM GP plunge freeze (Leica Microsystems) device. Grids were frozen in liquid ethane cooled by liquid nitrogen, at 90% relative humidity, and a chamber temperature of 4 °C. AYVV data was collected on an FEI Titan Krios (Astbury Biostructure Laboratory, University of Leeds) EM at 300 kV, using a total electron dose of 110 e-/Å^2^ and a magnification of 75,000 × . A total of 12,028 exposures were recorded using the EPU automated acquisition software on a FEI Falcon III direct electron detector, with a final object sampling of 1.065 Å/pixel. Each exposure movie had a total exposure of two seconds and contained 79 frames. AYVV has a strongly preferred orientation, with its 5-fold symmetry axis parallel to the ice layer. Particles with a view down the five-fold axis are not visualized. To increase the range of views, we collected 1810 movies with the stage tilted to 10° and 322 movies with the stage tilted to 20°.

### Image processing

A summary of image processing is shown in Supplementary Fig. 2. Image processing was carried out using the RELION 2.0/2.1 pipeline^33^. Drift-corrected averages of each movie were created using MOTIONCOR2 ^34^ and the contrast transfer function of each determined using gCTF^35^. Any images showing signs of significant astigmatism were discarded. Approximately 5000 particles were manually picked and classified using reference-free 2D classification^36^. The resulting 2D class average views were used as templates for automated particle picking using gAutomatch^37^ (see Supplementary Table 1 for particle numbers). Automated particle picking of particles on lacey carbon grids resulted in a large number of boxes picked on the edge of carbon (without AYVV particles). To classify these images away, 2D classification in RELION was used with CTF amplitude correction only performed from the first peak of each CTF onwards. Particles were further classified using several rounds of both reference-free 2D classification and 3D classification, with D5 symmetry imposed. A negative stain reconstruction was used filtered to ~60 Å resolution for the starting model. After each round, the best classes/class was taken to the next step of classification. Post-processing was employed to appropriately mask the model, estimate and correct for the B-factor of the maps^38^. The final resolution was determined using the ‘gold standard’ Fourier shell correlation (FSC = 0.143) criterion as 3.3 Å. Local resolution was estimated using the local resolution feature in RELION^33^.

### Refinement of atomic models

An X-ray structure of STNV (2BUK)^39^ was positioned within the AYVV density map at the equator (what was ultimately chain H, as this was the highest resolution region of the map; Supplementary Fig. 5) using rigid body fitting in UCSF Chimera^40^. Amino acid residues which did not fit into any density, particularly the loops which join the beta strands, were deleted and all remaining residues were changed to alanine using COOT^41^. The remaining backbone of the subunit was traced using COOT and the position of bulky amino acids was used to manually add the AYVV CP sequence^13^. Amino acids were built from residue 40–257. DNA nucleotides were then fitted into the density, also using COOT. The model was refined using three rounds of REFMAC^42^ with secondary structure restraints imposed using ProSMART^42^. After each refinement round, non-ideal rotamers, bond angles and Ramachandran outliers were improved using COOT. The resulting model of one subunit was symmetrized using Chimera and 11 subunits were fitted to represent one asymmetric unit, subunits A-K in one hemicapsid (Fig. 1). Amino acids 40–62 were removed for all subunits other than H and amino acids 55-62 were added for subunit I. This asymmetric unit was refined using the ‘real space refinement tool’ in COOT followed by three rounds in Phenix^43^ and four rounds in REFMAC^42^ with non-crystallographic symmetry constraints imposed. The quality of the asymmetric unit was assessed using MolProbity^44^ (Supplementary Table 1). Figures were generated using Chimera^40^, ChimeraX^45^ and PyMOL^46^.

### Data availability

Coordinates are deposited in the Protein Data Bank under accession code 6F2S. Cryo-EM reconstructions of the AYVV are deposited in the EM Data Bank under accession codes EMD-4174. All reagents and relevant data are available from the authors upon request.

## Electronic supplementary material

Supplementary Information

Description of Additional Supplementary Files

Supplementary Movie 1

Supplementary Movie 2

Supplementary Movie 3

Supplementary Movie 4

## References

[1] Saunders K, Bedford ID, Yahara T, Stanley J (2003). Aetiology: the earliest recorded plant virus disease. Nature.

[2] Harrison BD (1977). Plant-viruses with circular single-stranded-DNA. Nature.

[3] Saunders K (2000). A unique virus complex causes Ageratum yellow vein disease. Proc. Natl Acad. Sci. USA.

[4] Saunders K, Stanley J (1999). A nanovirus-like DNA component associated with yellow vein disease of Ageratum conyzoides: Evidence for interfamilial recombination between plant DNA viruses. Virology.

[5] Grigoras I (2009). Reconstitution of authentic nanovirus from multiple cloned DNAs. J. Virol..

[6] Gronenborn B (2004). Nanoviruses: genome organisation and protein function. Vet. Microbiol..

[7] Zhang W (2001). Structure of the maize streak virus geminate particle. Virology.

[8] Bottcher B, Unseld S, Ceulemans H, Russell RB, Jeske H (2004). Geminate structures of African cassava mosaic virus. J. Virol..

[9] Caspar DL, Klug A (1962). Physical principles in the construction of regular viruses. Cold Spring Harb. Symp. Quant. Biol..

[10] Perlmutter JD, Hagan MF (2015). Mechanisms of virus assembly. Annu. Rev. Phys. Chem..

[11] Prinsen P, van der Schoot P, Gelbart WM, Knobler CM (2010). Multishell structures of virus coat proteins. J. Phys. Chem. B.

[12] Hipp K, Grimm C, Jeske H, Bottcher B (2017). Near-atomic resolution structure of a plant geminivirus determined by electron cryomicroscopy. Structure.

[13] Tan PHN (1995). Genome organization of Ageratum yellow vein virus, a monopartite whitefly-transmitted geminivirus isolated from a common weed. J. Gen. Virol..

[14] Grimsley N, Hohn T, Davies JW, Hohn B (1987). Agrobacterium-mediated delivery of infectious maize streak virus into maize plants. Nature.

[15] Ford RJ (2013). Sequence-specific, RNA-protein interactions overcome electrostatic barriers preventing assembly of satellite tobacco necrosis virus coat protein. J. Mol. Biol..

[16] Harrison SC, Olson AJ, Schutt CE, Winkler FK, Bricogne G (1978). Tomato bushy stunt virus at 2.9 A resolution. Nature.

[17] Dent KC (2013). The asymmetric structure of an icosahedral virus bound to its receptor suggests a mechanism for genome release. Structure.

[18] Dai X (2017). In situ structures of the genome and genome-delivery apparatus in a single-stranded RNA virus. Nature.

[19] Stockley PG (2007). A simple, RNA-mediated allosteric switch controls the pathway to formation of a T=3 viral capsid. J. Mol. Biol..

[20] Liljas L (1982). Structure of satellite tobacco necrosis virus at 3.0 A resolution. J. Mol. Biol..

[21] Xie Q, Chapman MS (1996). Canine parvovirus capsid structure, analyzed at 2.9 Å resolution. J. Mol. Biol..

[22] Tuthill TJ (2009). Equine rhinitis A virus and its low pH empty particle: clues towards an aphthovirus entry mechanism?. PLoS Pathog..

[23] Bakker SE (2014). Limits of structural plasticity in a picornavirus capsid revealed by a massively expanded equine rhinitis A virus particle. J. Virol..

[24] Li CL, Wang JCY, Taylor MW, Zlotnick A (2012). In vitro assembly of an empty picornavirus capsid follows a dodecahedral path. J. Virol..

[25] Hesketh EL (2015). Mechanisms of assembly and genome packaging in an RNA virus revealed by highresolution cryo-EM. Nat. Commun..

[26] Kittelmann K, Jeske H (2008). Disassembly of African cassava mosaic virus. J. Gen. Virol..

[27] Chapman MS, Rossmann MG (1995). Single-stranded DNA–protein interactions in canine parvovirus. Structure.

[28] Sarker S (2016). Structural insights into the assembly and regulation of distinct viral capsid complexes. Nat. Commun..

[29] Tang L (2001). The structure of Pariacoto virus reveals a dodecahedral cage of duplex RNA. Nat. Struct. Biol..

[30] Saunders K, Lucy A, Stanley J (1991). DNA forms of the geminivirus African cassava mosaic virus consistent with a rolling circle mechanism of replication. Nucleic Acids Res..

[31] Klinkenberg FA, Ellwood S, Stanley J (1989). Fate of African cassava mosaic virus coat protein deletion mutants after agroinculation. J. Gen. Virol..

[32] Sainsbury F, Thuenemann EC, Lomonossof GP (2009). pEAQ: versatile expression vectors for easy and quick transient expression of heterologous proteins in plants. Plant. Biotechnol. J..

[33] Fernandez-Leiro R, Scheres SHW (2017). A pipeline approach to single-particle processing in RELION. Acta Crystallogr. D.

[34] Zheng SQ (2017). MotionCor2: anisotropic correction of beam-induced motion for improved cryo-electron microscopy. Nat. Methods.

[35] Zhang K (2016). Gctf: real-time CTF determination and correction. J. Struct. Biol..

[36] Scheres SH (2012). RELION: implementation of a Bayesian approach to cryo-EM structure determination. J. Struct. Biol..

[37] Urnvicius L (2015). The structure of the dynactin complex and its interaction with dynein. Science.

[38] Scheres SH, Chen S (2012). Prevention of overfitting in cryo-EM structure determination. Nat. Methods.

[39] Jones TA, Liljas L (1984). Structure of satellite tobacco necrosis virus after crystallographic refinement at 2.5- a resolution. J. Mol. Biol..

[40] Pettersen EF (2004). UCSF Chimera--a visualization system for exploratory research and analysis. J. Comput. Chem..

[41] Emsley P, Lohkamp B, Scott WG, Cowtan K (2010). Features and development of Coot. Acta Crystallogr. D.

[42] Brown A (2015). Tools for macromolecular model building and refinement into electron cryo-microscopy reconstructions. Acta Crystallogr. D.

[43] Adams PD (2010). PHENIX: a comprehensive Python-based system for macromolecular structure solution. Acta Crystallogr. D.

[44] Chen VB (2010). MolProbity: all-atom structure validation for macromolecular crystallography. Acta Crystallogr. D.

[45] Goddard TD (2017). UCSF ChimeraX: meeting modern challenges in visualization and analysis. Protein Sci..

[46] DeLano, W. L. The PyMOL Molecular Graphics System, Version 2.0 (Schrodinger, 2018).

