# Supplementary material for: The 3.3 Å structure of a plant geminivirus using cryo-EM
Source: Nat Commun. 2018 Jun 18;9:2369. doi: 10.1038/s41467-018-04793-6 (PMC6006435; doi:10.1038/s41467-018-04793-6)
Supplement: Supplementary file 1 — Supplementary Information [file 41467_2018_4793_MOESM1_ESM.pdf]

The 3.3Å structure of a plant geminivirus using cryo-EM

Hesketh *et al.*

Supplementary Figure 1.

***Ageratum yellow vein virus (AYVV) purification*.**

a. Schematic representation of AYVV's single stranded circular DNA genome, DNA A. DNA A contains all the proteins required for viral replication as well as the viral CP. b. Flowchart of AYVV preparation for cryo-EM. c. SDS-PAGE of purified fractions from Sucrose step gradient purification. The bands excised and analysed by MALDI-TOF are indicated with the red circle. d. The coat protein sequence of AYVV. MALDI-TOF analysis showed 51% protein sequence coverage (not shown) from purified AYVV particles. The peptides found are highlighted in red.

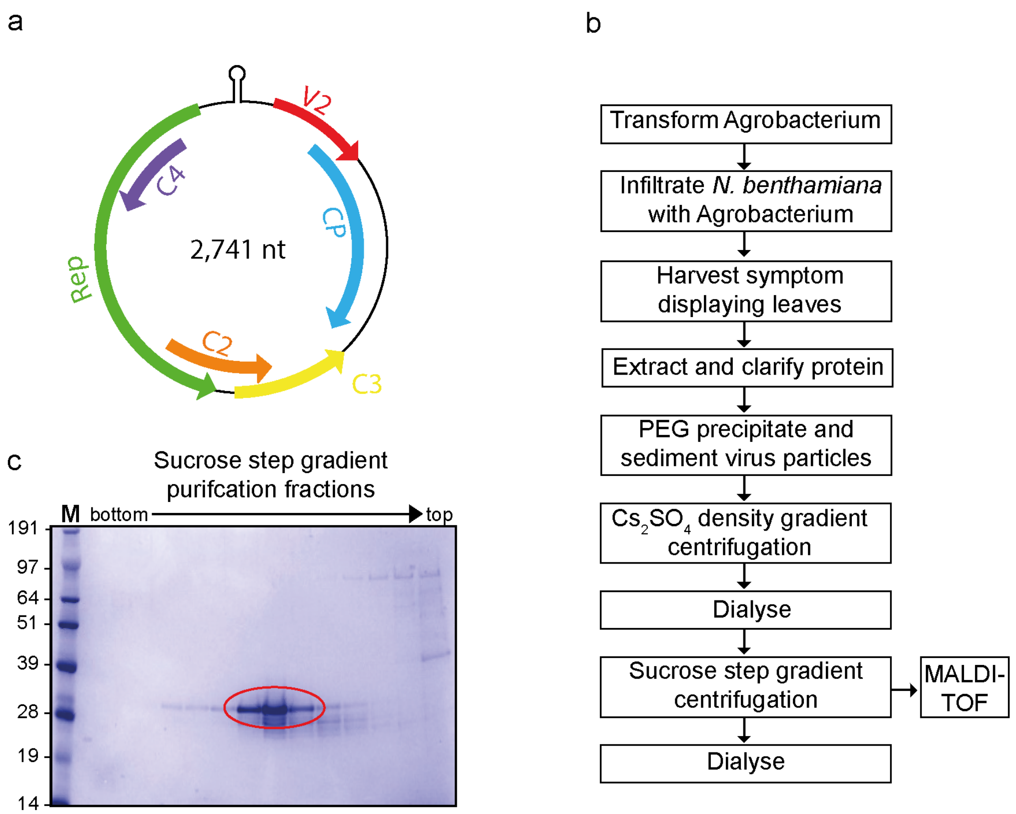

d

```
1   MSKRPADIVI STPASKVRRR LNFDTPVMSR AAAPTVLVTN RRRTWTNRPM
51  YRKPRLYRMY RTPDVPKGCE GPCKVQSYEQ RHDISHVGKV LCVSDVTRGN
101 GLTHRVGKRF CVKSVYVLGK IWMDENIKTK NHTNTVMFYL VRDRRPFGTA
151 MDFGQVFNMY DNEPSTATIK NDLRDRYQVL RKFTSTVTGG QYASKEQALV
201 KKFMKINNYV VYNHQEAAKY DNHTENALLL YMACTHASNP VYATLKIRIY
251 FYDSVQN
```

Supplementary Figure 2.

Cryo-EM raw data and refinement of AYVV.

a.) A representative cryo-EM micrograph of AYVV. Scale bar is 300 Å. b.) 2D class averages calculated using RELION2.0 c.) Left: The starting model used for a negative stain reconstruction of AYVV. Right: Negative stain reconstruction of AYVV at ~30 Å resolution. This map was used as a starting model for cryo-EM image processing. d.) Fourier Shell Correlation (FSC) curve of masked map, unmasked map and corrected map. The resolution reported here was according to the 0.143 criterion.

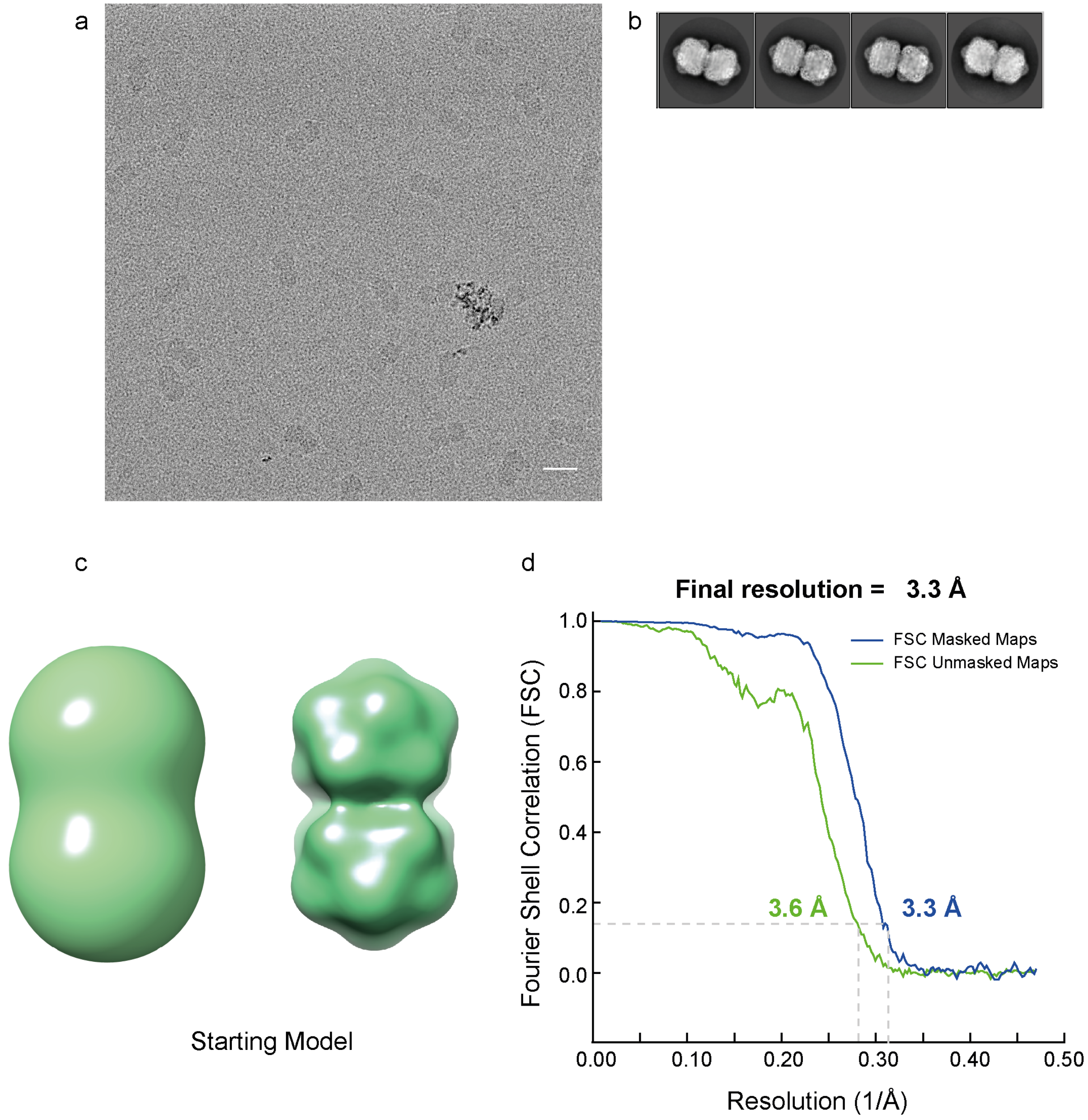

Supplementary Figure 3

Electrostatic surface representation of AYVV CP.

a.) An electrostatic surface representation of the CP for subunit A (representing the majority of the capsid) and subunits H and I (representing the subunits at the equator) using the Adaptive Poisson-Boltzmann Solver (APBS) plugin in PyMOL. The CP is shown with the interior of the capsid facing out from the page in the left column. This view is rotated 180° to show the exterior of the CP in the right column. The N -terminal region of the CP is highlighted with the black box, showing in subunits H and I this region is positively charged. b.) The first 62 amino acids of the CP are shown and the positively charged amino acids are highlighted to show this region is highly positively charged.

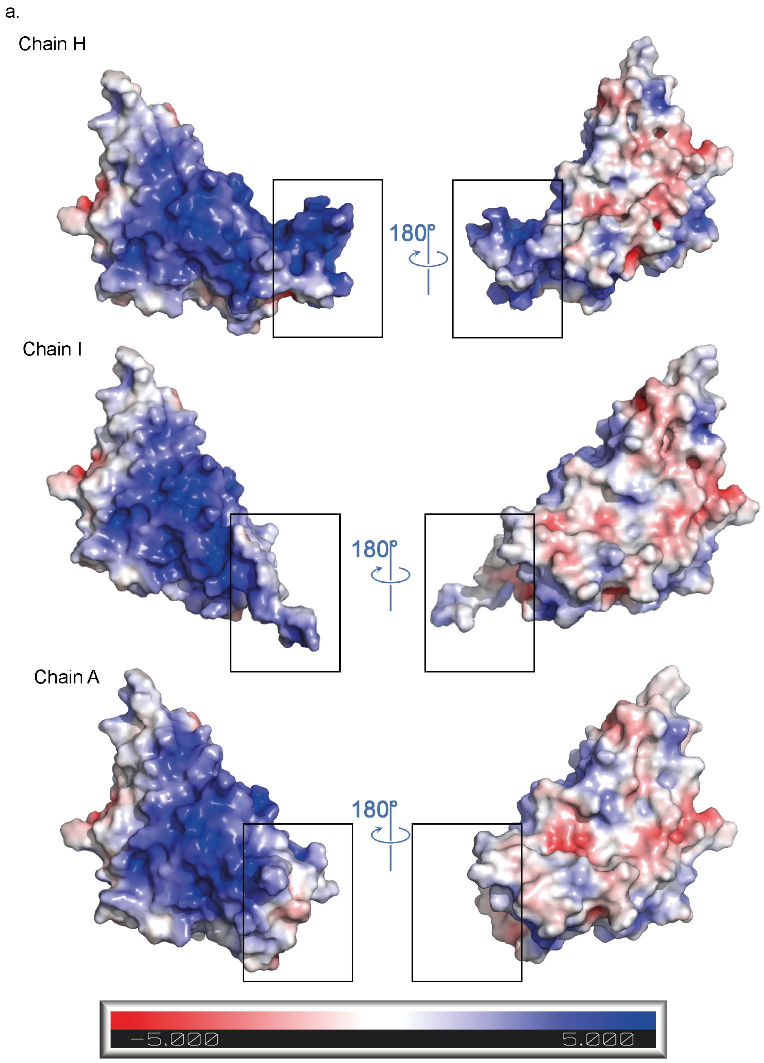

b.

```
       ++            + +++          +            +++    +
1    MSKRPADIVI STPASKVRRR LNFDTPVMSR AAAPTVLVTN RRRTWTNRPM
      ++ +  +   +
51   YRKPRLYRMY RT
```

Supplementary Figure 4

Polar residues involved in equator interface.

Space filling model of two asymmetric units of AYVV coloured as in Figure 1. Residues 214-216 are highlighted in orange in each subunit. In the isometric portions of the capsid these residues are located on the exterior surface of the capsid and therefore solvent exposed. At the equator (subunits H and I) this polar patch of residues becomes an integral part of the binding interface.

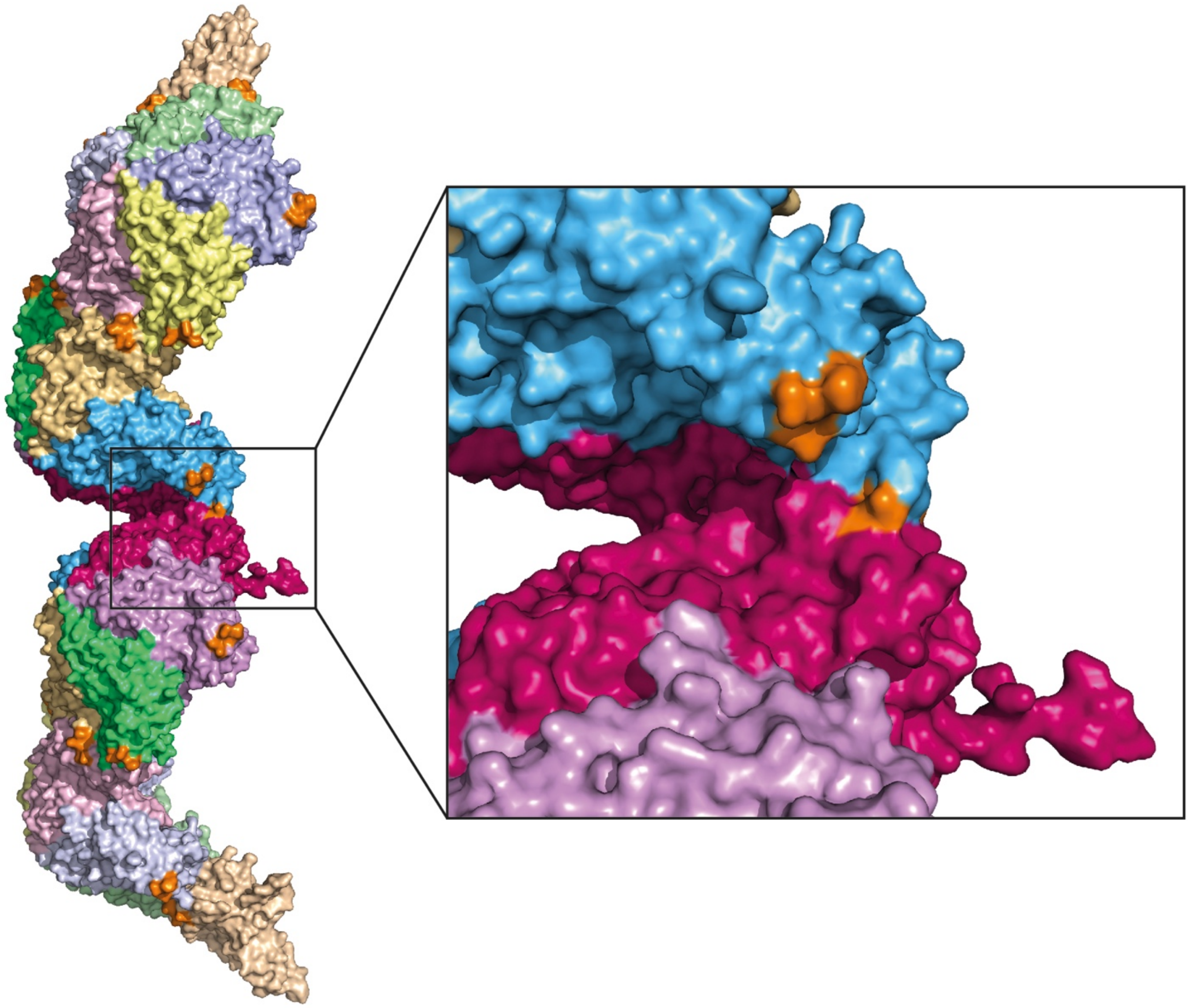

Supplementary Figure 5

Local resolution AYVV.

Exterior view of AYVV (left) coloured by local resolution using RELION2.0. The extremities of the map are the lowest resolution regions at 3.6 Å. The equator has the highest resolution regions at 3.1 Å suggesting this is the most stable region of the capsid. The interior view of AYVV (right) shows the majority of the interior of the capsid is at 3.2 Å resolution. A key is shown for reference.

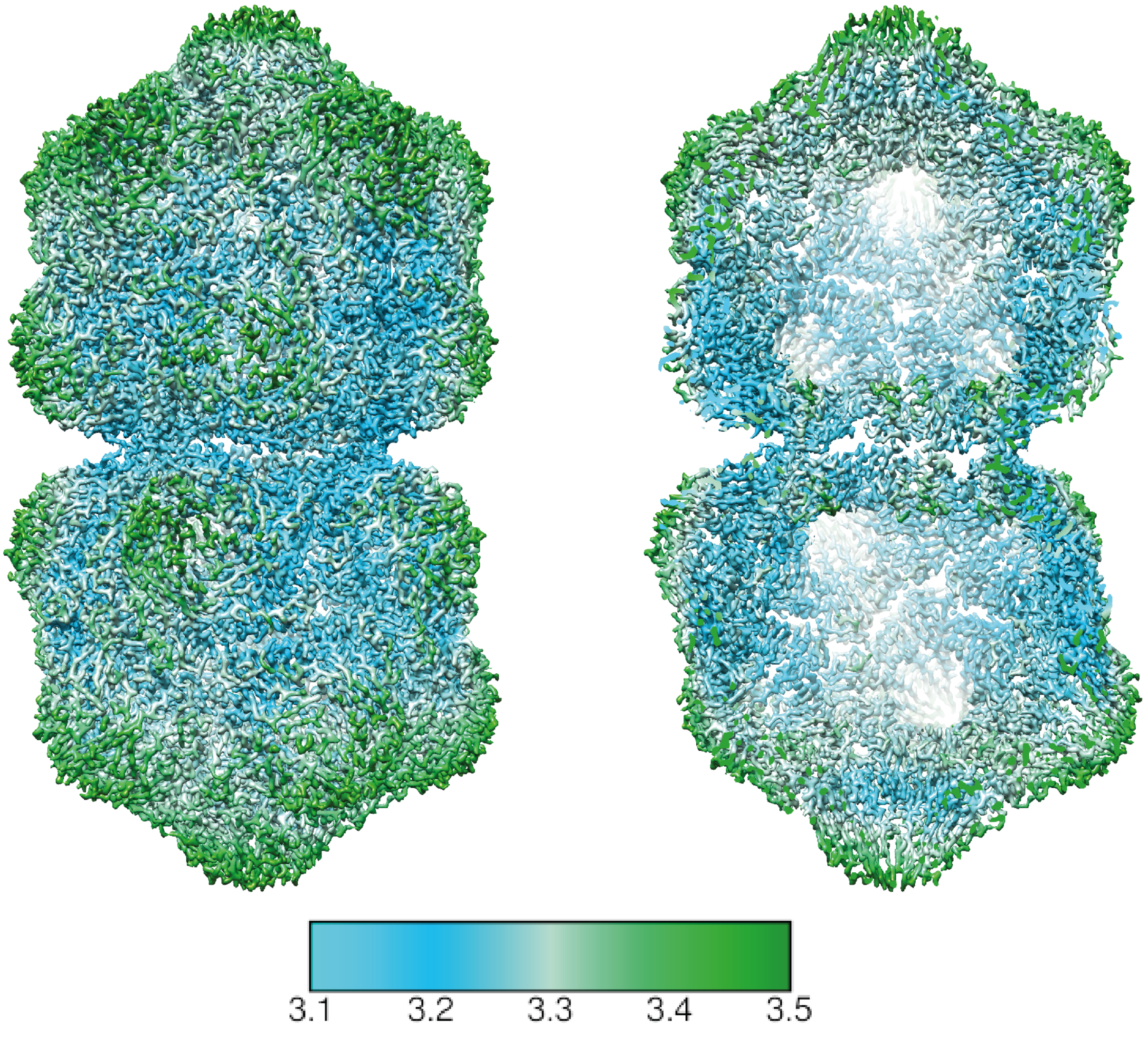

Supplementary Table 1.

Cryo-EM data collection, refinement and validation statistics.

	**AYVV:** EMD-4174, PDB 6F2S
**Data collection and processing**	
Magnification	75, 000 x
Voltage (kV)	300
Electron exposure (e–/Å^2)	110
Defocus range (μm)	-0.3 to -5.0
Pixel size (Å)	1.0651
Symmetry imposed	D5
Initial particle images (no.)	116, 240
Final particle images (no.)	64, 932
Map resolution (Å)	3.3
FSC threshold	0.143
Map resolution range (Å)	3.1 - 3.5
**Refinement**	
Initial model used (PDB code)	2BUK
Map sharpening *B* factor (Å^2)	-164.1
Model composition	
Non-hydrogen atoms	0
Protein residues	2, 176
Nuclei acid	76
R.m.s. deviations	
Bond lengths (Å)	0.0061
Bond angles (°)	1.18
Validation	
MolProbity score	2.20 (99th percentile)
Clashscore	19.78 (97th percentile)
Poor rotamers (%)	1.03%
Ramachandran plot	
Favored (%)	94.15
Allowed (%)	6.41
Disallowed (%)	0.56

Supplementary Table 2.

Oligonucleotide primers for molecular cloning and site-directed mutagenesis.

PRIMER	SEQUENCE
**KS37**	5’-GGGGACAAGTTTGTACAAAAAAGCAGGCTTAATGTCGAAGCGTCCCGCAGATATTG-3’
**KS38**	5’-GGGGACCACTTTGTACAAGAAAGCTGGGTTTAATTCTGAACAGAATCATAGA-3’
**KS125P**	5’-CTGTCCTCGTCACCAACGCAAGAAGGACATGGACCA-3’
**KS126P**	5’-TGGTCCATGTCCTTCTTGCGTTGGTGACGAGGACAG-3’
**KS127P**	5'-AAGGACATGGACCAACGCGCCCATGTACCGCAAG-3'
**KS128P**	5’-CTTGCGGTACATGGGCGCGTTGGTCCATGTCCTT-3’
**KS129P**	5'-GCAAGCCCAGACTGTACAGAGATTACAGAACCCCTGATGTGCC-3'
**KS130P**	5’-GGCACATCAGGGGTTCTGTAATCTCTGTACAGTCTGGGCTTGC-3’
