# Supplementary material for: The 3.3 Å structure of a plant geminivirus using cryo-EM
Source: Nat Commun. 2018 Jun 18;9:2369. doi: 10.1038/s41467-018-04793-6 (PMC6006435; doi:10.1038/s41467-018-04793-6)
Supplement: Supplementary file 2 — Description of Additional Supplementary Files [file 41467_2018_4793_MOESM2_ESM.pdf]

Descriptions of Additional Supplementary Files

File Name: Supplementary Movie 1: EM density to show single-stranded DNA binding – CD-type DNA.
Description: The exact view shown in Fig. 4b "rocking" to show the EM density (red mesh) and the atomic coordinates fitted within. CPs C and D shown and DNA stem loop N. i.e. DNA bound in the majority of the capsid (CPs A-G & J-K).

File Name: Supplementary Movie 2: EM density to show single-stranded DNA binding – HI-type DNA.
Description: The exact view shown in Fig. 4c "rocking" to show the EM density (red mesh) and the atomic coordinates fitted within. CPs H and I shown and the DNA stem loop S, i.e. DNA bound at the interface.

File Name: Supplementary Movie 3: Intimate interactions of DNA and CP – CD-type DNA.
Description: The exact view shown in Fig. 5a & b rotating to show the intimate interactions between the DNA and CPs C and D. Interactions are highlighted with green dashed lines.

File Name: Supplementary Movie 4: Intimate interactions of DNA and CP– HI-type DNA.
Description: The exact view shown in Fig. 5a & b rotating to show the intimate interactions between the DNA and CPs H and I. Interactions are highlighted with green dashed lines.
